# Evaluation of EMLA cream with microneedle patches in palatal anesthesia in children: a randomized controlled clinical trial

**DOI:** 10.1038/s41598-024-66212-9

**Published:** 2024-07-03

**Authors:** Farah M. Babakurd, Shadi K. Azzawi, Muaaz Alkhouli, Zuhair Al-Nerabieah

**Affiliations:** Pediatric Dentistry Department, Faculty of Dentistry, University of Damascus, Damascus, Syria

**Keywords:** Palatal injection, Pain, Anesthesia, Microneedle, EMLA, Health care, Medical research

## Abstract

Palatal injections are considered to be one of the most painful dental procedures. As a result, it was important to find alternatives to this painful injection to improve children's cooperation. The dental literature mentioned using EMLA cream as a possible alternative to conventional injections, but its anesthetic effect was debated. Therefore, it was valuable to research the impact of microneedle patches to enhance the effectiveness of this cream. The purpose of this randomized controlled clinical trial was to compare the effectiveness of different methods of anesthesia and pain levels in children aged 7–11 years. The study compared the use of EMLA cream, EMLA with microneedles, and conventional palatal injections. A total of 90 children were randomly assigned to three groups: Group 1 received conventional palatal anesthesia (control), Group 2 received EMLA cream only, and Group 3 received EMLA with microneedles. Pain levels were assessed using the FLACC and Wong-Baker scales at three different time points: T1(during anesthesia), T2(on palatal probing), and T3(during extraction). The FLACC scale revealed a significant difference in pain between groups only at T1 (*P* value = 0.000). It was found that the conventional palatal injection group had a higher pain level than the EMLA cream-only group and the group using microneedle patches with EMLA cream (*P* value = 0.000). However, the other groups did not show significant differences in pain levels during the anesthesia (*P* value  = 1.00). Similarly, the Wong-Baker scale also demonstrated a statistically significant difference in pain between groups only at T1 (*P* value  = 0.000). It was found that the conventional palatal injection group had a higher pain level than the EMLA cream-only group and the group using microneedle patches with EMLA cream (*P* value  = 0.000). However, the other groups did not show significant differences in pain levels during the anesthesia (*P* value  = 0.091). The study concludes that both EMLA cream alone and EMLA with microneedles can be used as an alternative to conventional palatal anesthesia for children.

## Introduction

The fear and anxiety caused by dental injections in children can lead to changes in their behavior and a loss of cooperation. Therefore, pain-free injections are crucial for successful pediatric dental treatment and to ensure a positive experience for the child in the future^1,2^. While traditional topical anesthetics like lignocaine and benzocaine have effectively reduced injection pain, they have not been as effective in reducing palatal injection pain^3^. This may be due to anatomical and histological differences, such as the thickness, keratinization, and fat of the palatal mucosa, in comparison to other areas of the oral mucosa^4^.

The hard palate is of medium thickness (310 µm) and is more keratinized to support its chewing function compared to the buccal mucosa (580 µm) and the floor of the oral mucosa (190 µm). The presence of ceramides in the water-impermeable keratinized epithelium makes the palatal mucosa less permeable to conventional surface agents^5–8^.

For palatal anesthesia, the topical anesthetic EMLA has been found to effectively penetrate the keratinized tissue due to its unique properties^9^. EMLA contains 2.5% lidocaine and 2.5% prilocaine, with a melting point of 17°C, allowing it to become liquid at an oral temperature^10,11^. Its relatively high pH (9.4) and lipophilic nature aid in its infiltration. It has a final concentration with a high water content that softens the keratin layer in the palate, allowing for a penetration depth of 5 mm^12,13^.

It was mentioned in the dental literature that EMLA cream could be used as a potential alternative to conventional injections^12^. The anesthetic effect of EMLA cream has been a topic of debate. Therefore, it is important to study the use of drug delivery systems, such as microneedle patches, to enhance the effectiveness of this cream^1,14^.

Drug delivery systems are effective at enhancing surface drugs' impact by reducing the mucosa's barrier properties through various mechanisms. These systems are typically divided into physical methods (such as pre-cooling, vibration, ion transport, and microneedle arrays) and chemical methods (including permeability enhancers, nanostructured carriers like liposomes, cyclodextrin, particle systems, polymeric nanoparticles, solid lipid nanoparticles, and nanostructured lipid carriers)^4^.

These physical delivery systems create micropores that temporarily breach the epithelial barrier of the palatine mucosa, enabling better permeability of surface drugs^15,16^.

Despite various developments and laboratory studies proving their effectiveness, microneedles are not yet commercially available as drug delivery systems in the oral cavity^7,17^. Therefore, for the first time in the current study, microneedle patches dissolved with EMLA cream are being used for palatal anesthesia.

This study aimed to evaluate the effectiveness of EMLA cream in providing anesthesia in the palate and to compare it with the application of EMLA using a microneedle patch for extracting upper primary teeth, without the need for a conventional local injection.

## Materials and methods

### Study design

A randomized, single-blinded, controlled clinical trial was conducted in the Department of Pediatric Dentistry at Damascus University from June to September 2021. The study was conducted according to the ethical standards outlined in the Declaration of Helsinki^18^. Ethical approval was obtained from the Ethical Research Committee at Damascus University No. 1999, dated May 20, 2021. This trial was registered at ClinicalTrials.gov with the following number: NCT05187494, 01/11/2022.

### Study sample

The sample size was calculated using G*Power software from Heinrich-Heine-Universität Düsseldorf, Germany, to assess changes in the main outcome, 'pain', using the FLACC score. The mean pain levels obtained by Cantekin et al. were utilized for this calculation^19^. Considering a power of 90% and a significance level of 5% in a superior clinical trial, the final sample was composed of 90 children, with 30 children per study group.

A total of 90 children between the ages of 7 and 11 were included in the study based on the following criteria:

1. Children who have never had a negative dental experience and who are cooperative and react positively or absolutely positively, according to the Frankl Behavioral Rating Scale^20^.
2. Healthy children without any nervous disorders or systemic diseases.
3. Children who have not received sedative drugs or pain medication in the last three hours before the procedure.
4. Children with upper primary teeth that are indicated for extraction.
5. Children with at least a third of the root visible on a radiograph.

The exclusion criteria were:

1. Allergy to anesthetic drugs.
2. Children with congenital or idiopathic methemoglobin.
3. Teeth with acute abscesses.

### Study groups

**Figure 1:**
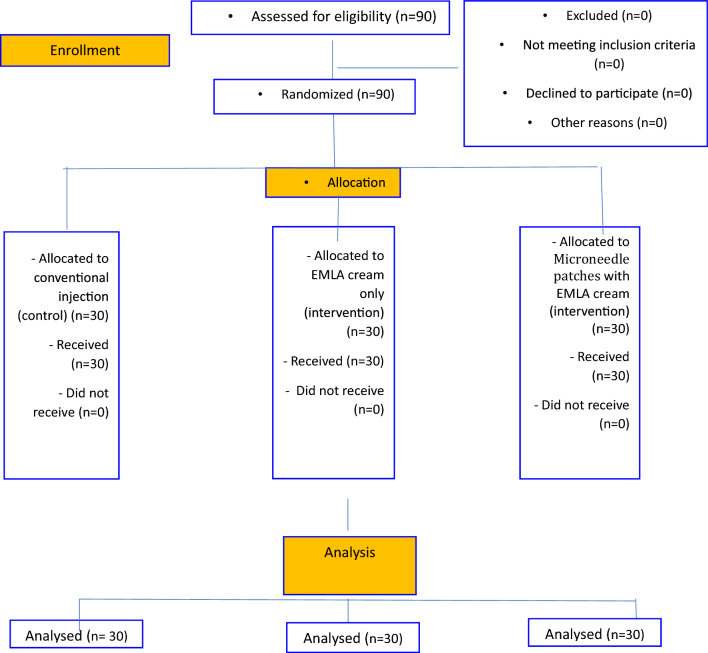
Consort flowchart of the study.

The sample was equally and randomly divided into three groups using the website www.randomalist.org. The groups are as follows: Group 1 received conventional local palatal injection (control group) for 30 children; Group 2 received only EMLA cream for 30 children; and Group 3 received microneedle patches dissolved with EMLA cream for 30 children. Figure 1 displays the CONSORT flowchart of the trial.

### Study intervention

The parents provided written informed consent for their children to participate in the study, following the previously mentioned criteria. They received a brief explanation of the procedures, potential benefits, and risks before providing their consent. The principal investigator (F.B.) performed clinical work and assessed pain and the effectiveness of palatal anesthesia for all children in the three groups. Pain assessment was done using both an objective scale (FLACC scale: Face, Legs, Activity, Cry, Consolability^21^) and a subjective scale (Wong-Baker Faces Scale, which ranges from a happy face at 0 to a crying face at 10^22^).

This assessment was conducted at the application, probing, and extraction stages for each of the studied groups.

### Group 1 (control group)

- Conventional palatal injection using lidocaine HCL 2% and Epinephrine 1:80,000 was given. The participant's reactions to the conventional injection were measured using the Wong-Baker Faces and FLACC scales.
- Subsequently, palatal probing was performed using a dental probe in contact with the bone^23^, and the effectiveness of the anesthesia was assessed by recording measures using the Wong-Baker Faces and FLACC scales.

The success criteria for the control group were defined as not requiring an additional palatal injection during probing.

### Group 2 (EMLA cream only)

- The palatal mucosa was dried with a cotton ball. Then, 0.2 g of EMLA cream 5% (Aspen, Sweden) was applied 1 mm away from the palatal gingival margin using cotton buds for 3 min within the application area of 14 × 14 mm. The mouth was kept open throughout the procedure, and saliva was controlled using a saliva ejector.

During this time, we used the Wong-Baker Faces and FLACC scales to measure the reaction to the applied substance.

b. After 3 min, the palatal mucosa was wiped with a dry cotton ball measuring 2 × 2 cm to stop the effect of the EMLA cream.

Following this, a palatal probe was performed with a dental probe in contact with the bone, and the reaction was recorded using the Wong-Baker Faces and FLACC scales.

Children who were unable to tolerate the pain during probing were excluded and given an additional traditional palatal injection to complete the extraction.

Group 3 (microneedle patches dissolved with EMLA cream): The palatal mucosa was dried with a 2 × 2 cm cotton ball. Then, 0.2 g of EMLA cream 5% was applied 1 mm away from the palatal gum margin using a microneedle patch measuring 14 × 14 mm and 0.25 microns (Rael Microneedle Patches, California) for 3 min. The mouth was kept closed throughout the procedure. During this time, we recorded the reactions to the corresponding substance using the Wong-Baker Faces and FLACC scales.

After 3 min, the patch was removed and the palatal mucosa was wiped with a dry 2 × 2 cm cotton ball to stop the effect of the EMLA cream. Then, we performed palatal probing with a dental probe in contact with the bone and recorded the same measures. Children who were unable to bear the pain during probing were excluded and given an additional traditional palatal injection to complete the extraction.

We first administered local anesthesia to the buccal area using 2% lidocaine HCL with 1:80,000 epinephrine (Korea) for all three groups. We then waited for five minutes to ensure adequate anesthesia^24^.

The extraction was carried out after this, and we recorded the same measures to assess the reaction during the extraction.

Blinding was not possible for each child or physician due to the differing treatment procedures in the control and experimental groups. However, the statistical analyst was blinded, and the decoding was only done after analyzing the results.

### Data analysis

Descriptive statistics and frequencies were calculated to present the results. Fisher’s exact tests were used to identify any statistical differences based on gender, age, type of primary tooth, and level of cooperation.

After conducting the Kolmogorov–Smirnov test to assess the normal distribution of the data, the results indicated that the data followed a normal distribution. Consequently, we used a one-way ANOVA test to determine if there was a statistically significant difference among the study groups on the FLACC scale.

The Kruskal–Wallis test was utilized to determine if there was a significant difference between the study groups in the Wong-Baker score. The significance level (*p*-value) for this study was set at 0.05. The statistical analysis was conducted using IBM SPSS software version 23 (IBM Corp., Armonk, USA).

## Results

In this study, 90 participants were enrolled, including 46 females and 44 males, with a mean age of 9.6 ± 1.29. Fisher's exact test showed no significant differences between the groups in terms of gender (*P* value  = 0.016), age (*P* value  = 0.029), type of primary tooth (*P* value  = 0.015), and level of cooperation (*P* value  = 0.024) of the treated children (Table 1).

**Table 1:** Demographic characteristics of the participants and fishermen's exact tests.

Groups	Gender N (%)		Age		Type of primary tooth N (%)					Level of cooperation N (%)	
	Male	Female	Mean	SD	Central Incisor	Lateral Incisor	Canine	1st Molar	2nd Molar	Positively	absolutely positively
Conventional	16 (53.3)	14 (46.7)	10.0	1.4	0 (0)	2 (6.7)	0 (0)	20 (66.7)	8 (26.7)	18 (60.0)	12 (40.0)
EMLA cream only	10 (33.3)	20 (66.7)	9.5	1.1	0 (0)	4 (13.3)	0 (0)	14 (46.7)	12 (40.0)	12 (40.0)	18 (60.0)
Microneedle patches dissolved with EMLA Cream	18 (60.0)	12 (40.0)	9.3	1.6	0 (0)	6 (20.0)	0 (0)	10 (33.3)	14 (46.7)	16 (53.3)	14 (46.7)
*P* Value †	0.016**	0.029**	0.015**	0.024**							

^†^Fisher’s exact tests, *Statistically significant, **Not statistically significant.

Each participant underwent assessment at three-time points: T1 (during anesthesia), T2 (during probing), and T3 (after extraction). The assessment utilized two methods (Wong-Baker and FLACC).

A one-way ANOVA test was conducted to analyze the difference in FLACC scores between the three groups at each time point. The results showed a statistically significant difference between the three groups at time point T1 (*P* value  = 0.000), as shown in Table 2.

**Table 2:** FLACC scores between the three groups at each time point.

Study phase	Groups	FLACC scores				P Value †
		0	1	2	3	
Application of Anaesthesia	Conventional	8	8	10	4	0.000*
	EMLA cream only	28	2	0	0	
	Microneedle patches dissolved with EMLA Cream	28	2	0	0	
Palatal probing	Conventional	24	6	0	0	0.13**
	EMLA cream only	26	2	2	0	
	Microneedle patches dissolved with EMLA Cream	30	0	0	0	
Extraction	Conventional	8	8	10	4	0.129**
	EMLA cream only	14	10	2	4	
	Microneedle patches dissolved with EMLA Cream	12	12	6	0	

^†^One-way ANOVA test, *Statistically significant **Not statistically significant.

The Mann–Whitney U test was used as a post hoc test to determine differences. It was concluded that in T1, the conventional palatal injection group had a higher pain level compared to the EMLA cream-only group and microneedle patches with EMLA cream (*P* value  = 0.000). However, the other groups did not show significant differences in pain levels during the anesthesia (*P* value  = 1.00), as shown in Table 3.

**Table 3:** Mann–Whitney U test for FLACC scores at the (application of anesthesia) time point.

Study phase	Groups	U value †	P Value †
Application of Anaesthesia	Conventional vs EMLA cream only	34	0.000*
	Conventional vs Microneedle patches dissolved with EMLA Cream	34	0.000*
	EMLA cream only vs Microneedle patches dissolved with EMLA Cream	112.5	1.00**

^†^Mann–Whitney U, *Statistically significant, **Not statistically significant.

The Kruskal–Wallis test was used to analyze the variation in the Wong-Baker scale among the three groups at each time point. A statistically significant difference was found between the three groups only at T1 (*P* value  = 0.000); see Table 4.

**Table 4:** Statistical analysis for Wong-Baker scale scores between the three groups at each time point.

Study phase	Groups	Chi-square	P Value †
T1	1	22.1	0.000*
	2		
	3		
T2	1	7.16	0.113**
	2		
	3		
T3	1	11.04	0.019**
	2		
	3		

^†^Kruskal–Wallis test, *statistically significant, **not statistically significant.

The Mann–Whitney U test was used as a post-hoc test to determine the differences. The results showed that in T1, the conventional palatal injection group had a higher pain level compared to the EMLA cream-only group and microneedle patches with EMLA cream (*P* value  = 0.000). However, the other groups did not show significant differences in pain levels during the anesthesia (*P* value = 0.091); see Table 5.

**Table 5:** Post hoc test for Wong-Baker scale scores.

Study phase	Groups	U value †	P Value †
T1	1 vs 2	20	0.000*
	1 vs 3	42	0.000*
	2 vs 3	75	0.091**

^†^Mann–Whitney U, *Statistically significant, **Not statistically significant.

## Discussion

Palatal injections are known to be among the most painful types of injections inside the mouth. This is because the palatal mucosa is firmly attached to the periosteum, requiring the application of positive pressure during administration^25,26^, Its thick, keratinized layer resists the effects of the surface anesthetic^27,28^, and these tissues are prone to local complications such as burning, ulceration, and tissue swelling, which may lead to the reactivation of latent viruses such as herpes simplex virus as well as bruising^29^.

Therefore, a search was conducted to find alternatives to the palatal injection in modern dentistry^30,31^.

Based on the similarity of the penetration depth of EMLA cream and the depth of insertion of the palatine needle (5 mm) to reach the superficial palatine nerve^32,33^, it is possible to replace painful palatine injections. In a study by Munshi et al. (2001), EMLA cream was applied with a gauze piece for 10 min on both the vestibular and palatine sides when extracting upper jaw teeth for children aged 4 to 13 years, eliminating the need for a needle. However, the success of this method was limited in cases of loose teeth and remnants of roots^34^. Therefore, for comprehensive treatment in all cases, the focus was narrowed down to the palatine side, considering the anatomical location of the superior alveolar nerve, which requires a needle insertion depth of 15 mm to reach it^32,33^. This limits the use of EMLA cream on the vestibular side due to its 5 mm penetration depth. Chugh et al. (2021) conducted a study on the application of EMLA 5% cream using a gauze piece for 10 min limited to the palatine side, combined with an injection of 2% lidocaine with 1:80,000 adrenaline in the vestibular side for cases of upper jaw tooth extraction in adults, and achieved a satisfactory level of palatal anesthesia^13^.

Considering that the length of treatment is linked to the children's behavior rather than the type of local anesthesia or the complexity of the treatment^35^, Jamali Z (2018) proposed that each phase of treatment should take no more than 5 min to engage the child effectively^36^.

Based on the above information, along with the study by Barcohana N. (2003) which found similar efficiency of EMLA cream when applied for different times (3, 5, and 10 min)^37^, the decision was made to choose an application time of 3 min instead of 10 min as an alternative to palatal injection.

A variety of external factors contribute to changes in the oral mucosa, which can temporarily and painlessly alter its permeability^17,38,39^.

An example of this is microneedle patches, which were proposed in the 1990s as an effective physical drug delivery system^40^.

Solid and hollow microneedles were initially patented by Gastrel and Place in 1971. In 1975, Paul received a patent for coated microneedles, while dissolving microneedles were developed in the 2000s^41^.

Several laboratory studies have shown that microneedles can create pores to enhance the penetration of topical medications^17^.

One of the downsides of solid microneedles was that they were made from non-biocompatible materials, and required a two-stage process of creating micro-pores and then applying the surface anesthetic separately^42^.

Augusto G. in 2019 conducted a clinical study on solid microneedles. The study concluded that microneedling treatment of the palatine mucosa increased the effectiveness of surface anesthesia of EMLA cream when applied 5 min before palatal injection^43^.

(Kang YH, 2017) demonstrated the potential use of microneedles as a new and effective method for conscious sedation in children. The study focused on utilizing hollow microneedles, which are solid needles with a drug reservoir^44^.

The two-stage process used for solid microneedles was avoided by using coated microneedles, which simplified the process to just one step. This involved a group of solid microneedles coated with a water-soluble, bioacceptable drug. However, the composition still contained solid microneedles, making it not completely acceptable. Additionally, the amount of anesthetic in the coating was small but sufficient for vaccine administration^44^.

In a study by Ma Y (2015), it was mentioned that drug-coated microneedles are an attractive tool that can uniformly and effectively deliver drugs to localized oral cancers, thus impacting the treatment of oral cancer patients^45^.

To make these needles biocompatible, fully dissolved microneedles are made of water-soluble or biocompatible polymers. The drug is completely dissolved in the matrix of the needles, and this process is also limited to a single step^44^. This method has been used in many treatments, including aphthous stomatitis^46^.

A specialized tank was designed to circumvent the limited anesthetic supply, but the challenging manufacturing process and slow drug release led to the development of dissolved microneedles using centrifugal technology^15^.

Despite the various advancements in microneedle technology and the positive results from laboratory studies, these microneedles are still unavailable for commercial use as drug delivery systems in the oral cavity^7,17^. This is partly due to the challenges in manufacturing them^47^. In this study, microneedles that dissolve were used for the first time to treat the palate as an alternative to the commonly used palatine injection. The palatine injection Despite the various advancements in microneedle technology and the positive results from laboratory studies, these microneedles are still unavailable for commercial use as drug delivery systems in the oral cavity. This is partly due to the challenges in manufacturing them. In this study, microneedles that dissolve were used for the first time to treat the palate as an alternative to the commonly used palatine injection. The microneedles are typically used to treat skin conditions by stimulating collagen and elastin through micropores^48,49^, and the oral mucosa's structure is similar to that of the skin^38^.

The oral mucosa has a higher permeability range of 4–4000 compared to the skin, which allows these microneedle patches to penetrate the palatine keratinized layer, dissolve in the interstitial fluid, and create micropores^15,38^.

It was possible to use the adhesive-tight frame surrounding the microneedle label to apply EMLA cream, addressing issues like low viscosity and bitter taste, and completing the procedure in one step^3,9^.

By employing microneedles as drug delivery systems, we reduced the application time of EMLA cream to just 3 min. This method is non-toxic because the microneedles are made of biocompatible materials, and the amount of 5% EMLA cream used is minimal and does not reach toxic levels.

According to the study by Pickers ER (1997), a 30-min EMLA application to the mucous membrane resulted in a safe plasma concentration of prilocaine (223 ng/ml) and lidocaine (418 ng/ml), much lower than the known toxic levels of both prilocaine (4.4 µg/ml) and lidocaine (0.6 µg/ml)^50^.

In previous reports, it has been mentioned that EMLA cream may cause local paleness, edema, burning sensation, and itching^51^, and rarely methemoglobinemia in children^52^. However, none of these symptoms were observed in our study. Our findings align with those of the Daly et al. study, as no negative occurrences were reported by the examiner who visually examined the oral soft tissues. Additionally, no disadvantages were reported regarding the patch needles^53^.

The study focused on children aged 7–11 years because this age group is the most cooperative and capable of understanding the procedures and providing clear reactions. The cases selected for the study involved tooth extraction, as this is a particularly painful procedure that often causes fear and anxiety^54^. The success of the study is expected to provide insight into improving other dental procedures as well.

This study found that there was a difference in pain levels when using the FLACC scale and the Wong-Baker scale to assess children in T1. However, there were no differences between the three groups during probing and extraction. This could be because both EMLA alone and EMLA with a microneedle effectively numbed the palatal tissue. In contrast, conventional anesthesia caused more pain than the other two methods of palatal anesthesia.

Limitations of this study include the inability to blind examiners to the type of anesthesia used and the absence of physiological measures to assess pain, as they could be affected by the anesthetic solution itself. Future studies could explore the use of EMLA cream with microneedle patches in different age groups and for other dental procedures. Additionally, investigating the cost-effectiveness of this approach compared to traditional palatal injections could be beneficial.

## Conclusions

In conclusion, using microneedle patches dissolved in EMLA cream proved to be an effective alternative to traditional palatal injections for anesthesia in children aged 7–11 years undergoing tooth extraction. The results showed that using EMLA cream with microneedle patches resulted in a significant reduction in pain compared to traditional palatal injections.

Additionally, the use of EMLA cream with microneedle patches was found to be safe and well-tolerated by the children in the study. Furthermore, the results of this study support previous research on the use of EMLA cream for anesthesia, but with the added benefit of microneedle patches for improved delivery and absorption.

This approach has the potential to reduce the fear and anxiety associated with traditional palatal injections in pediatric dental patients, leading to a more positive dental experience.

## Consent to participate

Human subjects: Consent was obtained or waived by all participants in this study. Approval number 1999 was issued by the Faculty of Dentistry, Damascus University, on May 20, 2021. This trial was registered at ClinicalTrials.gov on January 11, 2022, with the document number NCT05187494. Animal subjects: The study did not involve animal subjects or tissue. Conflicts of interest: All authors have confirmed that no financial support was received from any organization for the submitted work. They have also declared that they have no financial relationships at present or within the previous three years with any organizations that might be interested in the submitted work. Furthermore, the authors have declared that no other relationships or activities could appear to have influenced the submitted work.

## Data Availability

Data are available on reasonable request and stored as de-identified participant data. Don't hesitate to get in touch with babakrdfarah@gmail.com to request the data.
